# The latent system factors that influence antimicrobial use and governance in healthcare: a scoping review of high-income health systems

**DOI:** 10.1016/j.eclinm.2025.103520

**Published:** 2025-09-19

**Authors:** Olivia Lounsbury, Marta Ortega Vega, Lauren Hookham, Jane O'Hara, Natalie Sanford, Kelly Williams, Andrew J. Brent, Helen Higham

**Affiliations:** a Nuffield Department of Clinical Neurosciences, University of Oxford, Oxford, UK; b Florence Nightingale Faculty of Nursing, Midwifery & Palliative Care, Methodologies Division, King’s College London, London, UK; c Department of Global Health and Infection, Brighton and Sussex Medical School, Brighton, UK; d The Healthcare Improvement Studies (THIS) Institute, University of Cambridge, Cambridge, UK; e National Institute for Health and Care Research (NIHR) Yorkshire & Humber Patient Safety Research Collaboration (PSRC), Bradford, UK; f Patient Safety and Quality Department, The Johns Hopkins Hospital, Baltimore, USA; g Chief Medical Officer & Professor of Infectious Diseases & General Medicine, Oxford University Hospitals NHS Foundation Trust, Oxford, UK; h Visiting Professor of Infectious Diseases & General Medicine, Nuffield Department of Medicine, University of Oxford, Oxford, UK; i Anaesthetics, Oxford University Hospitals NHS Foundation Trust, Oxford, UK; j Oxford Simulation, Teaching, and Research Centre (OxSTaR), University of Oxford, Oxford, UK

**Keywords:** Antimicrobial resistance, Antibiotic resistance, Antimicrobial stewardship, Antimicrobial, Antibiotic, Sociotechnical system, Systems approaches, Safety governance, Healthcare safety, Sepsis

## Abstract

**Background:**

Inappropriate antimicrobial use accelerates antimicrobial resistance (AMR), creating tensions between antimicrobial stewardship and timely treatment. Despite global calls to action, gaps between recommendations and practice persist, largely due to system factors shaping clinical work. This review examines how systems approaches have been applied to the use and governance of antimicrobials.

**Methods:**

This scoping review followed PRISMA-ScR. Eligible sources included empirical studies, reviews, protocols, theses/dissertations, and improvement projects across healthcare settings in high-income countries. Studies required explicit use of systems approaches. EMBASE, MEDLINE, and CINAHL were searched in November 2024. Grey literature was added in February 2025. Search was updated in July 2025. Three researchers extracted data on study characteristics, systems approaches, and organisational implications. Review was preregistered (https://osf.io/p3xsf).

**Findings:**

25 articles were ultimately included. Seven focused on acute care and 18 leveraged interviews. Among studies involving participants (n = 18), 12 involved only frontline level participants, four involved participants from different organisational levels with a focus on frontline settings, and two focused on management settings. Implications of the use of systems approaches were identified across three organisational levels: micro (patient care), meso (management), and macro (leadership).

**Interpretation:**

This is the first review to map the implications of systems approaches for AMR at different organisational levels. Findings suggest gaps in the application of systems approaches at levels most responsible for strategic decisions and implementation. Given recent criticism about safety management and calls for strong AMS leadership, this gap represents a missed improvement opportunity. Future research should explore how alignment toward AMS is impacted due to system factors within and between organisational levels.

**Funding:**

Supported by INEOS Oxbridge Initiative on Antimicrobial Resistance (Project number E4R00010).

**Research in context**

**Evidence before this study**

EMBASE, MEDLINE, and CINAHL were searched from inception to November 12, 2024 with grey literature searches conducted on 15 February 2025 and an updated search conducted on 9 July 2025 prior to publication. Articles were included if a primary aim of the study was to understand the system factors influencing antimicrobial use or governance in healthcare organisations in US, UK, European Union, Australia, New Zealand, Canada, or Norway. Search terms included combinations of system-related search terms (e.g., “system design”, “system factors”) and antimicrobial-related search terms (e.g., “antimicrobial stewardship”). Eligible sources included empirical studies, reviews, protocols, theses/dissertations, and quality improvement projects. Studies were included if they explicitly stated that a systems approach was used. Risk of bias was not assessed, as this was a scoping review focused on mapping the breadth of existing evidence, rather than evaluating study quality. While previous work has explored behavioural, policy, and implementation factors that influence the use of antimicrobials, no reviews have synthesised how systems approaches have been applied across organisational levels.

**Added value of this study**

This is the first review to examine how systems approaches have been used to understand antimicrobial use and governance at micro (frontline), meso (management), and macro (leadership) levels. It identifies a striking imbalance in that most research that has applied systems approaches has focused on frontline challenges. Though this is essential, there has been minimal exploration of system factors that influence antimicrobial stewardship (AMS) efforts at higher organisational levels.

**Implications of all the available evidence**

This review highlights the need to recognise that conditions at the micro level are influenced by system factors that impact work at higher organisational levels. There is a need to better understand the system factors that impact each organisational level, and the interactions between organisational levels, to design more aligned systems toward AMS goals.

## Introduction

Early treatment of infections is important to prevent harmful sequelae including sepsis. However, inappropriate use of antimicrobials exposes patients to avoidable harms, such as *Clostridioides difficile*, and contributes to antimicrobial resistance (AMR), in which pathogens become less responsive to antimicrobials due to overuse.^1^, ^2^, ^3^ Leading healthcare authorities globally have called for immediate action to minimise AMR and antimicrobial stewardship (AMS) programmes have emerged to support clinicians in promoting judicious antimicrobial use.^4^^,^^5^ However, given that clinicians must often make antimicrobial prescribing decisions amid uncertainty, there is a persistent difficulty in overcoming the tension between reducing antimicrobial exposure and early appropriate treatment of infections while coping with other pressures of clinical work.^6^

Despite decades of research and stewardship initiatives, guideline discordant prescribing persists.^7^^,^^8^ Examples include unnecessary treatment of asymptomatic bacteriuria, prolonged antibiotic courses for community-acquired pneumonia, or the overuse of broad spectrum antibiotics (BSA) when narrower spectrum antibiotics are available. In the case of suspected sepsis, for example, it has been suggested that unnecessary empiric therapy can contribute to higher mortality, underscoring the need for judicious use of BSA.^9^^,^^10^ Clinicians have also highlighted the need for more careful use of BSA for infection management more broadly; surveys of physicians and pharmacists found that decreasing inappropriate BSA use was perceived to be the top opportunity to reduce AMR.^11^ There are extensive resources to guide both the starting of appropriate antimicrobials and the revision of the antimicrobial treatment plan.^12^, ^13^, ^14^, ^15^, ^16^ However, evidence shows that antibiotic use continues to increase in UK hospitals, with an estimated 5% increase in antibiotic prescribing from 2019 to 2023.^17^ While rising antibiotic use may partly reflect changing patient demographics and increased comorbidity and complexity, the persistence of guideline-discordant practices suggests there may be more complexity in the relationship between guidance and practice that should be explored to inform policy and practice.^18^

This gap between guidance and practice, referred to as the “work-as-imagined (WAI), work-as-done (WAD)” gap, is influenced by several system factors that emerge within clinical work, contribute to workflow challenges, and drive the need for clinicians to adapt in real time.^19^, ^20^, ^21^ Examples of system factors include efficiency pressures, misalignments between demand and capacity, ambiguities in care, or inadequate technologies.^20^, ^21^, ^22^, ^23^ Blaming clinicians for non-compliance is too often the approach taken, which fails to recognise the litany of intersecting system factors that influenced, and will continue to influence, patient outcomes if unaddressed.^24^ A systems approach considers how the dynamic interactions between tools, technologies, people, tasks, and socio-organisational factors influence work and sheds light on the valuable human adaptations that preserve patient safety despite system challenges.^25^, ^26^, ^27^ Frameworks such as the Systems Engineering Initiative for Patient Safety (SEIPS), Systems Ambiguity Framework, and Dynamic Safety Model, have been developed to depict how systems influence outcomes, such as AMR.^22^^,^^23^^,^^28^ The potential for systems approaches to shed light on the compounded effects of various system factors has been previously recognised by the infectious diseases community as imperative for advancements in AMS.^24^ However, little is known about how systems approaches have been previously used to understand this WAI, WAD gap related to antimicrobial use.

To design work processes that support, rather than hinder, stewardship goals, understanding the problem through systems approaches beyond the clinical level in isolation is a prerequisite. While systems approaches have been used to study other frontline clinical contexts, system factors impact work at every level of an organisation, including those responsible for oversight and governance.^29^^,^^30^ For example, data used to inform safety priorities at the middle management level is partially taken from incident reporting systems, which myopically capture information only when something goes wrong. If incident reporting systems encourage frontline reporting of only *incidents* (e.g., sepsis), rather than *harm* with or without an incident (e.g., AMR), AMR may not be identified as an organisational priority for which resources should be allocated. Organisational oversight and day-to-day clinical practice intersect regularly. Therefore, uncovering system factors at both frontline and governance levels will inform how multi-level work impacts antimicrobial use holistically within healthcare organisations.^30^

The question for this review is “How have systems approaches been used to understand antimicrobial use and governance in healthcare organisations?” The objectives of the review are to.

• Map existing literature to understand how systems approaches have been applied
• Describe implications for improvement based on the use of systems approaches at different levels of healthcare organisations

## Methods

The PRISMA-ScR Checklist and guidance from the Joanna Briggs Institute were used to inform the methods.^31^^,^^32^ This review was pre-registered (osf.io/p3xsf).^33^

### Search strategy and selection criteria

Eligibility criteria were developed based on previous literature and this review's aims. Few articles were identified during the preliminary search that focused specifically on systems approaches to the AMR-sepsis tension. Therefore, we expanded eligibility criteria to include antimicrobial use more broadly. Articles from all healthcare settings in the US, UK, European Union, Australia, New Zealand, Canada, and Norway were included. These countries were specifically selected because their economic, legal, and cultural healthcare characteristics are likely to similarly influence the systems within which antibiotics are used. Additionally, these geographies have established formal AMR collaborations and maintain advanced AMR surveillance systems.^34^

Articles that explicitly used systems approaches to understand how antimicrobials were used or governed were included. A “systems approach” is challenging to define due to the discipline's eclectic origins. We defined a systems approach as a way of understanding work that recognises how multiple elements interact to impact processes and outcomes, characterised by interactions between system factors, emergence, and feedback loops.^26^^,^^27^ For an article to be included, authors must have made explicit reference to systems approaches, which is similar to strategies used in previous reviews.^35^ See Appendix 1 for inclusion criteria.

The Ovid Embase, Ovid MEDLINE, and CINAHL EBSCOhost search strategies were executed via EBSCOhost on November 12, 2024 based on librarian recommendation. Grey literature searching included backward citation tracking and searching of thesis/dissertation portals. See Appendix 2 for the original database and grey literature search strategies. The same searches were reconducted on July 9, 2025 to ensure the results were as up to date as possible prior to publication.

Results were imported into Covidence for deduplication and screening. 10% of articles were screened by a combination of two of three blinded reviewers (OL, LH, MOV). All reviewers discussed the results of initial screening, reconciled results, and clarified criteria. The remaining articles were screened independently by two of three blinded reviewers. Disagreements were discussed before full text screening.

Data from the articles were extracted by two independent reviewers. The setting, publication date, country, article type, how systems approaches were used, and implications across organisational levels for the future of AMR work were extracted from every article. The extraction template was constructed according to project aims and previous literature and was piloted with the first 10% of articles. The data extracted for all articles was discussed between reviewers and a final output for each was generated.

### Data analysis

Quantitative synthesis consisted of categorical representation of healthcare settings, geographical areas, and study designs. Qualitative analysis consisted of descriptive and thematic analysis of the use of systems approaches and its implications. Two researchers independently extracted qualitative data from each article. The group constructed preliminary themes and refined the approach through constant comparison.

The author-reported implications from their use of systems approaches were thematically analysed and three organisational levels were constructed: Micro, meso, and macro. The extraction template was then updated to continue extraction related to these levels. The “micro” level referred to frontline staff delivering clinical care. The “meso” level referred to those involved in management/governance roles (“middle management”). The “macro” level referred to senior leaders involved in organisational decision making. Regardless of the participants involved, implications could have been identified at any level (e.g., frontline staff could have identified macro level implications). Thematic analysis was performed to identify sub-themes within each level. We aimed to understand the meaning of the results for future AMS improvement work, not to develop a taxonomy of the system factors that impact antimicrobial use. Therefore, we focused on implications, rather than results.

### Role of the Funding source

The funder had no role in study design or execution.

## Results

**Fig. 1:**
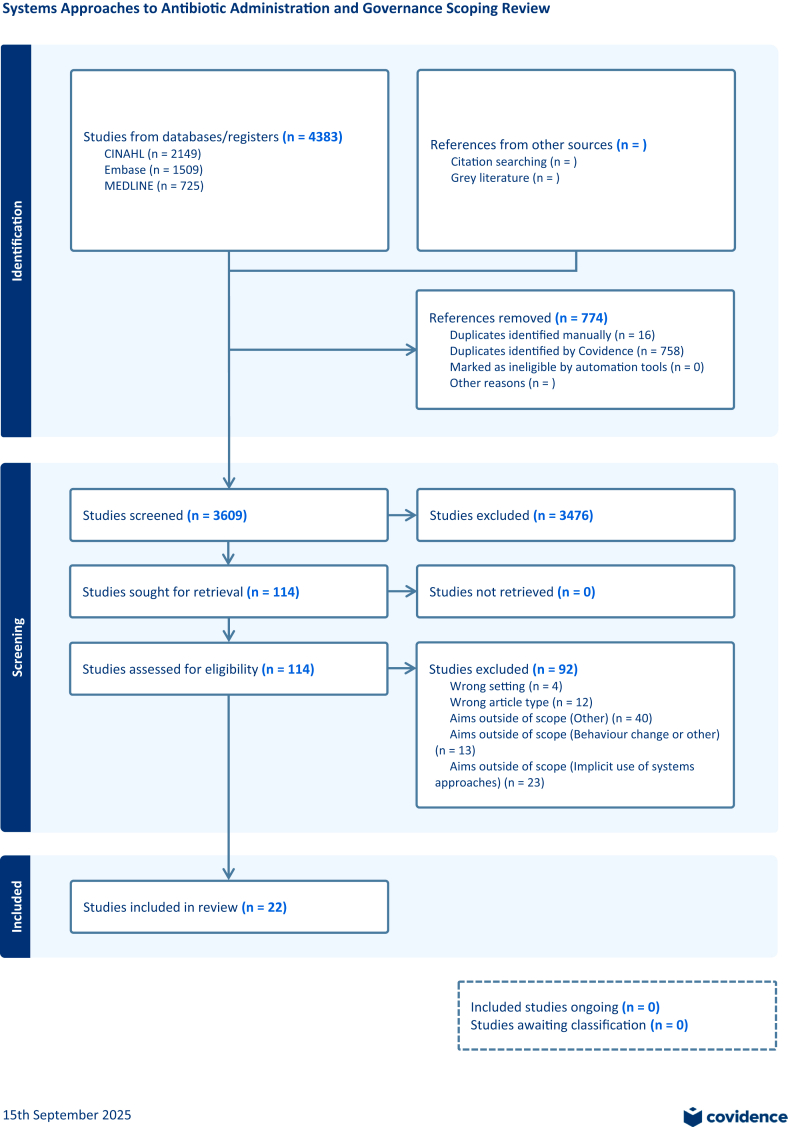
**Study selection.**

4385 articles were identified during the November 12, 2024 database searching. Duplicates were removed, 3611 were screened, and 116 were moved to full text review. See PRISMA chart (Fig. 1). Reasons for exclusion included wrong setting, wrong article type, or aims outside of scope (Behaviour change or other (n = 15), implicit use of systems approaches (n = 23), or other (n = 40)). See the Supplementary Materials for more detail on exclusion reasons. The search was repeated on Jul 9, 2025 to ensure all relevant articles were captured before publication. During this repeat search, one additional article by Van Dort and colleagues (2025) was identified, bringing the total number of included articles to 25. PRISMA guidance encourages researchers to transparently report the processes used to select data from multiple reports:^36^ All results, including protocols and reviews, were treated as individual papers and results were reported separately.

The first included study was published in 2010 with an increase in studies during and after COVID-19 (n = 18 from 2020 to July 2025). Most studies were conducted in the US (n = 15) with fewer in other areas, including the UK (n = 3), Australia (n = 4), European Union (n = 2), Sri Lanka (n = 1), South Africa (n = 1), East Asia (n = 1), and Canada (n = 1). Though Sri Lanka, South Africa, and East Asia geographies were not part of the inclusion criteria, these areas were included in the ‘geographic area’ count because they were included in studies also conducted in the UK. UK-specific data were extracted where possible to align with inclusion criteria. The most common settings were acute care (medical, surgical, n = 7), outpatient (n = 5), and emergency/urgent care (n = 5). Two (n = 2) studies were conducted at management/leadership levels. See Table 1.

**Table 1:** Characteristics of included studies.

Study	Date	Country	Article type	Setting	Participants
McNeil	2017	UK	Thesis/dissertation	Acute care	Consultants, registrars, foundation doctors, physician's associates, pharmacists, nurses; Members of Association of Scottish Antimicrobial Pharmacists
Tarrant	2021	UK, South Africa, Sri Lanka	Original research	Acute care	Hospital prescribers with experience of working with medical and surgical patients
Leyenaar	2019	US	Original research	Acute care; Emergency/urgent care	Collaborative participants across the US, including pediatric hospitalists, nurses, pharmacists, emergency physicians
Catho	2020	EU, Switzerland	Original research	Acute care	Physicians, including residents, senior fellows, attendings
McLellan	2016	UK	Original research	Acute care	Junior doctors
Krukas	2020	US	Original research	Acute care	No participants; Secondary data
Knobloch	2021	US	Original research	Outpatient	US Department of Veterans Affairs (VA) nurse practitioners, Veterans
Legenza	2023	US	Original research	Outpatient; Emergency/urgent care	Physician, nurse practitioner, physician assistant
Keller	2018	English language only	Review	Outpatient	No participants
Bugeja	2020	Australia, USA, Canada, EU, East Asia	Review	Outpatient; Long-term care facilities	No participants
Kianmehr	2020	US	Original research	Outpatient	No participants; Secondary data
Aagaard	2010	US	Original research	Emergency/urgent care	Nurses, ED staff, project leaders, nurse managers, quality improvement officers
Valmadrid	2021	US	Original research	Emergency/urgent care	Nurses and physicians working in long term care facilities or EDs
Pulia	2022	US	Original research	Emergency/urgent care	Emergency physicians
Ramly	2020	US	Original research	Long-term care facilities	Nursing home leadership (nursing directors or managers, infection control practitioners), nurses, prescribers
Ramly	2021	US	Original research	Long-term care facilities	Nursing home leadership (nursing directors or managers, infection control practitioners), nurses, prescribers
Katz	2017	English language only	Review	Long-term care facilities	No participants
Broom	2021	Australia	Original research	Management/leadership	Hospital executives
Hernandez	2024	US	Original research	Management/leadership	Clinical implementation coordinators (clinicians who acted as site liaisons and interacted with the intervention as a clinician. Coordinators filled informal roles within their implementation role, such as planner and facilitator)
Safdar	2021	US	Protocol	Critical care	Frontline alert users
Carayon	2021	US	Original research	Critical care	The two leaders (An AMS physician and an AMS pharmacist) responsible for the design and implementation of a fluoroquinolone pre-authorisation intervention
VanDort	2024	Australia	Original research	Pharmacy/AMS teams	Doctors and pharmacists involved in AMS tasks
Gagnon	2014	US	Quality improvement case study	Pharmacy/AMS teams	No participants
Hughes	2024	Any	Review	Any	No participants
Van Dort	2025	Australia	Original research	Acute care	AMS team members from varied professional backgrounds including pharmacy and infectious diseases

“Acute care” included medical and surgical care. “Outpatient” included office, outpatient parenteral antimicrobial therapy, family care, and infusion centres. “Long-term care facilities” encompassed skilled nursing facilities and nursing homes.

ED, Emergency department.

Eighteen studies involved participants. Twelve involved only frontline level participants with a focus on frontline settings, four involved a combination of frontline level participants and participants from other organisational levels with a focus on frontline settings,^37^, ^38^, ^39^, ^40^ and two involved participants from other organisational levels with a focus on management/leadership settings.^41^^,^^42^ The remainder (n = 7) were reviews or studies that used secondary data. See Table 1.

Interviews and focus groups were used more than other data collection approaches (n = 18). Observations (n = 5), reviews (n = 4), document review (n = 4), surveys (n = 2), quality improvement (n = 2), secondary data (n = 3), instant reporting (n = 1), incident reporting (n = 1), and implementation diaries (n = 1) were also used. Studies could have leveraged more than one data collection method. Based on the results as stated by the authors, AMS practices are shaped by a dynamic interplay of individual (e.g., fear, autonomy), social (e.g., hierarchies, team norms), and structural factors (e.g., diagnostic access, institutional incentives). The effectiveness of AMS interventions were found to vary by context, with leadership and culture, feedback, and staff stability and relationships facilitating optimal antimicrobial use. See Appendix 3 for more detail on the results of each article.

Articles were only included if systems approaches were explicitly discussed in the article. 84% (n = 21) of studies' designs were informed by systems approaches. 16% (n = 4) of studies' use of systems approaches was for results interpretation.^41^^,^^43^, ^44^, ^45^ Systems Engineering Initiative for Patient Safety (SEIPS) was the most commonly used systems-based framework (n = 12) and was used for several purposes, including underpinning study rationale, informing study design, and conducting data collection and analysis.^40^^,^^42^^,^^46^, ^47^, ^48^, ^49^, ^50^, ^51^, ^52^, ^53^, ^54^, ^55^ Other approaches included Rosen's modeling relation,^37^ Pettigrew's Receptive Contexts to Organisational Change,^56^ Flottorp framework,^57^ System Dynamics Simulation Model,^58^ complexity theory,^35^ and Cognitive Task Analysis coupled with Operations Sequencing Diagramming.^59^ Others explicitly provided their own description of a systems approach based on existing literature and used this description to guide their analysis.^38^^,^^39^^,^^60^ See Appendix 3 for more detail on how systems approaches were used and the results reported by the authors.

The frontline level was most often the focus in the articles reviewed, which is similar to what has been found previously.^46^ Key implications for the future of AMR work at the micro level were categorised into use and usefulness of standard operating procedures, adaptations and interdependencies, context, communication and trust, the role of technology, signals of activity and commitment, and the role of education. There were examples of people-based activities (e.g., making commitments to antimicrobial stewardship visible) positively influencing AMS.^39^^,^^40^ In other cases, there were negative implications associated with people-based activities, particularly re-education, as this was viewed to be insufficient alone without system change.^44^^,^^49^ Some authors stated that the usefulness of any intervention is contingent on the understanding of the system in which it will be situated, which will vary between settings (e.g., medical versus surgical), infrastructure (e.g., whether the AMS team is remote or on-site), and workflows (e.g., frequency of rounds).^37^^,^^43^

Little work using systems approaches has been conducted at the meso level with meso level participants, despite their role in implementation, coordination between departments, and integration of new standards. Key implications for the future of AMR work at the meso level were categorised into metrics and data reporting, coordination between projects, role of the “implementer”, and the representation of perspectives.Though there were fewer implications reported at the meso level, this may not be because there are fewer implications for those involved in management or governance roles, but may suggest that this level's role in AMR has been understudied. Indeed, several studies commented on the need to further explore the nuance involved in the role of ‘middle managers’ and how their role contributes to AMS.^42^^,^^46^^,^^54^ Other studies identified meso-level implications but were primarily focused on frontline settings and involved frontline participants.^39^^,^^46^^,^^54^^,^^56^^,^^57^^,^^60^

Similarly, work using systems approaches at the macro-level was minimal. Key implications for the future of AMR work at the macro level were categorised into language, external influences on the organisation, and organisational structure and reporting. The macro-level implications were characterised by a vague understanding of what ‘good’ antimicrobial stewardship leadership looks like.^41^^,^^54^ Broom and colleagues (2021) argued that ‘good’ antimicrobial stewardship leadership is at odds with current governance structures.^41^ Even in studies examining the nature of antibiotic-related safety event reports, ‘errors’ consisted of dosing and administration errors but there was no mention of errors contributing to AMR.^44^ Similarly, the relatively minimal data at the macro level may not necessarily suggest that there are fewer implications for those in senior or executive positions, but may suggest that those at this level of the organisation have been understudied in the context of AMR. See Table 2.

**Table 2:** A summary of the author-reported implications for the future of antimicrobial resistance (AMR) work by organisational level.

Level	Description	Author-reported implications for the future of AMR work
Micro	Frontline staff involved in delivering clinical care	Use and usefulness of standard operating procedures • Some guidelines were associated with a perceived loss of autonomy and critical thinking skills^57^ • Appropriate prescribing not always reflective of guideline-concordant “best” practice^45^^,^^53^ • Conflicting organisational guidance (e.g., for sepsis versus for AMS) influenced practice^56^ Adaptations and interdependencies • Actions were taken specifically to avoid facing unnecessary complexity^43^ • Providers most often used general websites to support antibiotic decision making (e.g., UpToDate) and did not use local AMR data sources (e.g., on the intranet) even though providers knew about the local resources^59^ • Feedback loops to providers were deemed helpful to adapt practice,^58^ specifically increasing follow up touch points, which reduced the inclination to prescribe^53^ • Nurses informally took on role as steward^51^ • Tensions between care domains (e.g., satisfaction/patient-centredness versus safety),^39^^,^^52^^,^^53^^,^^58^ cost and effectiveness,^59^ efficiency and certainty,^43^ and opportunities and threats of new clinical decision support systems^57^ Context • The context within which the intervention was implemented significantly influenced the intervention's impact^37^^,^^46^ • Workflow comparisons enabled benchmarking between sites and helped to identify context-specific features^37^ • There was a need for a surgery-specific AMS team due to surgery-specific cultural and organisational factors^48^ Communication and trust • Nurses relied on middlemen to communicate information to doctors^38^ • Difficult for providers to follow up with patients for a change in antibiotic when lab results come back^59^ • Warm handoffs between hospitals and outpatient facilities recommended to improve antimicrobial communication^51^ • Trust between clinicians and patients key facilitator of AMS, which requires time^52^ • Relationships built on trust between the infectious diseases teams and frontline teams were instrumental for success of AMS programmes^49^ The role of technology • Telemedicine can enable involvement of remote antimicrobial stewardship professionals but should be complemented by other organisational interventions^40^ • Knowledge gaps may be best addressed through embedded clinical decision support systems that are *easily* accessible^53^ • Digital information sharing tools were not fit for purpose which resulted in workarounds using paper based tools^38^ • Understanding of context/normal work and clarifying the purpose of any new digital tool is imperative before implementation^48^ Signals of activity and commitment • Champions facilitated buy in^56^ • Local leadership, visible commitment, and regular communication and data sharing were necessary^39^^,^^50^ • Funding of AMS positions amplified the attention on AMS because it seemed leaders were “serious”^48^ The role of education • The need for ongoing education may in itself justify a pressing need for human factors approaches and that the high number of educational interventions may suggest constraints on the extent to which system redesign approaches are feasible^40^^,^^44^ • Education is necessary but insufficient without system change or without taking into account local constraints^35^^,^^40^^,^^50^ • Education is necessary but regular communication and feedback are required to break patterns^54^
Meso	Those involved in clinical or non-clinical management or governance roles	Metrics and data reporting • System barriers related to the submission of organisational data to benchmarking and collaborative groups^60^ • Too many required measures from different entities, which leads to prioritisation of some metrics over others^39^ Coordination between projects • Lack of coordination between projects, competing improvement priorities, and challenges with dissemination of information contributed to poor performance overall and were barriers to interdepartmental change^56^ • Multidisciplinary meetings with multiple departments more effective than pharmacy department alone^42^ Role of the “implementer” • Intervention “implementers” are significantly influenced by organizational characteristics^41^ • Presence of implementation support personnel for new clinical decision support system (CDSS) facilitated uptake^57^ Representation of perspectives • Characteristics of other non-clinical healthcare team members and the factors that impact their role related to antimicrobial use should be studied^46^
Macro	Senior or executive leaders	Language • Our notion of ‘good’ - as a society, in hospitals- informs our governance systems and is often at odds with the interventions needed for meaningful change for long term challenges such as AMR. There is a need to redefine what ‘good’ AMS leadership looks like, particularly in the face of challenges that require long term solutions for which typical micro-improvements will not suffice^24^ • Further clarification on "antimicrobial management" in the governance context is needed^60^ External influences on the organisation • Have not been thoroughly studied^46^ • High national priority on sepsis overshadows antimicrobial resistance prevention^43^ • Organisations and leaders are dependent on directives from the state^24^ • Managers are incentivised to maintain the AMS status quo^24^ Organisational structure and reporting • Where the AMS team ‘sits’ within the organisation can have an impact on its effectiveness. For example, if the AMS team ‘sits’ within infectious diseases, AMS is seen as a priority specific to infectious diseases, rather than an organisational safety priority^48^ • The lack of a centralised AMS governance structure across the hospital resulted in inconsistent practice^49^ • Departmental structure (e.g., the paediatric emergency department being part of the emergency medicine division, rather than the paediatric division) made information sharing and interdepartmental coordination with paediatric acute care floors challenging^56^ • Importance of alignment of organisational infrastructure with the antimicrobial stewardship priority (e.g., audits, feedback, aligned incentives)^43^ • Standardisation of guidelines/interventions across the organisational to prevent piecemeal uptake and changes in standards when a patient moves wards^50^ • Regular communication, explanation of rationale, and leadership commitment across levels is imperative for successful implementation^50^

## Discussion

To the authors’ knowledge, this is the first review to construct an understanding of the implications of systems approaches for AMR at micro, meso, and macro organisational levels.

The limited application of systems approaches beyond the micro level suggests a gap in understanding how organisational alignment toward the AMS goal-or lack thereof-emerges across levels due to system factors within and between levels. Systems approaches at the micro level are imperative. However, frontline challenges are influenced by the institutionally-sanctioned context within which they occur and are often downstream consequences of system factors at higher organisational levels.^61^ Given recent critiques about the impact of poor middle management in patient safety more broadly,^62^ applying systems approaches to the meso-organisational level with meso-level participants is even more important. For the purposes of our study, those at the meso level were defined as “Those involved in clinical or non-clinical management or governance roles”. Based on the results of the included studies, this group varied significantly and could range from nurse managers on one ward to governance specialists to those on a secondment focused on implementing a new practice change. This was the most nebulous group compared with micro and macro, which likely reflects the reality in practice where complex organisational structures may contribute to wide variation in roles, reporting structures, and departmental responsibilities at the middle management level. Middle managers play a crucial, but underexplored role in bridging organisational priorities with frontline contexts, meaning that the system factors that impact their work are ill-defined. The absence of literature on the use of systems approaches at the meso-organisational level related to the AMR “invisible pandemic”^63^ suggests a blackhole in our understanding and missed opportunity for AMS improvement.

In spite of numerous calls for strong AMS leadership, few studies examined system factors that impact macro level work. Instead, macro level factors were often vaguely alluded to, rather than robustly explored. System factors at the macro level, including reporting structures (e.g., configuration of departments), organisational priorities (e.g., interdependencies between goals), governance approaches (e.g., feedback to staff), and staffing models (e.g., rotation schedules) may reinforce alignments or drive misalignments that impact AMS progress. For example, McLellan and colleagues (2016) found that resident doctors were often ideally positioned to identify several system barriers, but did not have the seniority, permanent position, or resourcing capacity to address these problems.^45^

Whilst many articles uncovered systems factors that impact antimicrobial use, without an explicit focus, these factors risk being treated as facilitators and barriers to specific actions (e.g., reducing BSA use), rather than part of a complex interplay between influences at different levels. This is similar to findings from other reviews.^35^^,^^49^^,^^50^ The integrative review by Katz and colleagues (2017) stated that none of their included studies used human factors approaches.^49^ This may reflect a lack of understanding of what a systems approach is or, if it is understood, potential barriers to its use in practice. See Appendix 4 for information about articles that were excluded at the full text stage due to the lack of an explicit systems approach. If system factors are identified opportunistically without a clear understanding of their interconnectedness (e.g., via systems approaches), building cohesive strategies to address them becomes difficult, resulting in patchwork approaches to improvement.

Our findings suggest that even studies that have explicitly applied systems approaches still uncover areas in which behaviour change, rather than system change, interventions are best suited. As has been previously suggested, in some contexts, system improvements are not a substitute for behaviour change, professional responsibility, or education.^61^ Likewise, in other contexts, behaviour change interventions cannot be seen as a solution to a systems problem. This suggests the need to move away from the limiting dichotomy of behaviour versus the system to the behaviours within the system.^61^ In behaviour science, for example, insight into people's knowledge, attitudes, motivations, and biases can be leveraged to design interventions that influence human behaviour, such as incentive pathways.^64^^,^^65^ Others suggest that knowledge, motivations, and biases must be complemented with systems change for the desired action to occur.^44^^,^^49^ Choosing the right intervention (whether a behavioural intervention, system redesign, or both) depends on an accurate diagnosis of what drives the observed behaviours within the system. Understanding these dynamics can reveal insights for AMS improvement that neither systems thinking nor behavioural science could fully uncover in isolation.

Overarchingly, this review revealed a general need to focus on the interplay between all organisational levels to drive AMS improvements. Key points at the macro level include reframing AMS as a core priority, not a specialist concern. Some articles showed that AMS often ‘sits’ within the infectious diseases group, which may limit its perceived relevance to broader organisational goals. Positioning AMS within broader quality and safety governance structures may make it more visible, particularly as many quality and safety governance structures are increasingly resourcing programmes to evaluate the management of patients with sepsis. A first step might include, for example, recommending that quality improvement teams audit where AMS sits within the organisational charts and better align stewardship with other system-wide goals (e.g., readmission rates, throughput, specialty surgery volumes).

In terms of future research, three key points emerged. First, there is a need to clarify what ‘good’ AMS leadership looks like. Future researchers may consider designing a Delphi study, for example, to understand the similarities and differences in what ‘good’ AMS leadership looks like to different stakeholders. Second, there is a need to understand how to measure AMS more meaningfully. The existing literature suggests that there are several contextual points that influence AMS practice, such as frontline relationships and adaptations to circumvent ineffective processes, that are not likely to be captured in our current AMS measurement structures. However, monitoring these contextual points is key to understanding how the system works in practice, not just how it is intended to work on paper. Future researchers should consider how AMS can be more holistically measured to anticipate, respond to, and learn from AMS risks. Previous work has suggested that patient harm rates should be just one component of several in a robust safety measurement strategy.^66^ Other components include reliability (How reliable are the clinical systems and processes?), sensitivity to operations (Is there a deep awareness of day to day functioning and variability?), anticipation and preparedness (How are AMS risks anticipated?), and integration and learning (How is data used to respond and improve?). Future research to tailor these principles for AMS specifically may help to reveal the inner workings of a complex AMS system to complement what is already known about AMS outcomes. Finally, the variety of seemingly informal workarounds (e.g., relying on nurses as middlemen, not using local AMS resources but seeking out general point of care tools such as UpToDate) suggests that there may be a difference between the mechanisms that are perceived to be valuable to the users and those perceived to be valuable by the organisation. Future research is needed to understand what mechanisms are seen as valuable to support AMS work and which are fulfilled to signal virtue.

This review's limitations should be acknowledged. Relevant articles may have been excluded if the use of systems approaches was unclear in the abstract. Understanding the interactions between system factors was difficult due to heterogeneity in how studies were reported, which has been stated previously.^26^^,^^35^^,^^50^ We regularly revisited the question “How explicit is explicit enough to be considered a ‘systems approach’?” which pushed us to include only articles that explicitly stated its use. Finally, this article focused on geographies with well-established healthcare systems and similar structural, legal, and cultural characteristics. While the inclusion of similar geographies enabled a degree of comparability, a similar exploration should be conducted in other geographies.

## Contributors

OL contributed to the conceptualisation, organisation, design, development, and writing of the manuscript. LH and MOV contributed to conceptualisation, initial screening, full text review, and writing. LH contributed clinical subject matter expertise. NS contributed research subject matter expertise and to the writing of the manuscript. KW contributed clinical and improvement subject matter expertise and to the writing of the manuscript. AJB, JO, and HH served as senior clinical, research, and improvement experts, guided conceptualisation and final manuscript development, and secured resources to facilitate the success of this project. OL, LH, and MOV had access to the underlying data. All authors have read and approved the final version of the manuscript.

## Data sharing statement

All data extracted from the articles included in this study will be made available to others on request to the corresponding author.

## Declaration of Interests

This work was supported by the INEOS Oxbridge Initiative on Antimicrobial Resistance though the funder was not involved in the development of this manuscript. AJB is a Member of Board of Hospital Trust. JOH is supported by the National Institute for Health and Care Research (NIHR) Yorkshire & Humber Patient Safety Research Collaboration (PSRC). The views expressed are those of the authors and not necessarily NIHR or the Department of Health and Social Care. Authors do not have any other interests to declare.
