# Supplementary material for: The latent system factors that influence antimicrobial use and governance in healthcare: a scoping review of high-income health systems
Source: eClinicalMedicine. 2025 Sep 19;89:103520. doi: 10.1016/j.eclinm.2025.103520 (PMC12495420; doi:10.1016/j.eclinm.2025.103520)
Supplement: Multimedia component 1 [file mmc1.docx]

**Appendix 1: Inclusion and exclusion criteria**

|  | **Inclusion Criteria** | **Exclusion Criteria** |
| --- | --- | --- |
| **Population** | All patient populations |  |
| **Setting** | Any healthcare delivery organisation including but not limited to hospitals, urgent care, primary care, mental health, and pharmacies | National public health organisations (e.g., the Centers for Disease Control and Prevention) or non-healthcare settings (e.g., animal health) |
| **Concept** | Systems approaches defined as a way of understanding work that recognises how multiple elements interact to impact processes and outcomes, characterised by interactions between system factors, emergence, and feedback loops | Behaviour change; Clinical education; Implementation science without the explicit mention of systems approaches |
| **Aim of the Articles** | Articles were included if a primary aim of the study was to understand the system factors influencing either the   - Prescription, dispensing, administration, monitoring, adjustment, or discontinuation of antimicrobials in frontline work or - Organisational leadership or governance of antimicrobial use (e.g., organisational goals, policies, stewardship programmes, or institutional oversight)   Authors must have explicitly stated that a systems approach was used. | Articles were excluded if   - System factors were incidental findings - The study focused only on infection control or sepsis without a primary focus on antimicrobials (e.g., *C. diff* prevention through hand hygiene) - The study focused on highly protocolised procedures (e.g., antimicrobial administration within 60 minutes of an order, routine prophylaxis) |
| **Article type** | Peer-reviewed, empirical, original research articles; Reviews; Protocol papers; Theses; Dissertations; Published quality improvement (QI) projects | Other grey literature such as viewpoints; Governmental reports; Letters to the editor; Conference transcripts; Conference posters or presentations; Theoretical papers; Books |
| **Study design** | No restrictions on study design |  |
| **Country** | US, UK, European Union, Australia, New Zealand, Canada, Norway |  |
| **Language** | Any language |  |
| **Date Range** | Articles published on any date prior to the day of the first search (November 12, 2024) or refreshed search (July 9, 2025) |  |

**Appendix 2: Search Strategy**

MEDLINE, EMBASE, and CINAHL were searched based on librarian recommendation of the databases that would be relevant for the review’s aims. The final search strategies were formally iterated nine times with relevant key papers used to “test” the robustness of the search strategy. The final search strategies for MEDLINE and EMBASE based on these iterations and librarian recommendations are provided below.

**Databases**

MEDLINE (725 results as of Nov 12, 2024)

Medline (Ovid MEDLINE® Epub Ahead of Print, In-Process & Other Non-Indexed Citations, Ovid MEDLINE® Daily and Ovid MEDLINE®) 1946 to present

1 exp Drug Resistance, Microbial/ 192904

2 ("antimicrobial resistance" or "antimicrobial stewardship").ti,ab,kf. 54244

3 amr.ti,ab,kf. 12091

4 exp Anti-Bacterial Agents/ 862914

5 antibiotic*.ti,ab,kf. 474386

6 1 or 2 or 3 or 4 or 5 1149181

7 exp United States/ 1515977

8 exp United Kingdom/ 403376

9 exp Australia/ 182088

10 exp New Zealand/ 46366

11 exp Canada/ 191877

12 exp Europe/ 1612340

13 exp Norway/ 44291

14 ("united states" or "USA" or "United Kingdom" or "UK" or "Britain" or "England" or "Scotland" or "Wales" or "Northern Ireland" or "National Health Service" or "NHS" or "Australia*" or "Canada*" or "New Zealand" or "Europe" or "Norway").mp. 2520552

15 7 or 8 or 9 or 10 or 11 or 12 or 13 or 14 4026514

16 exp Ergonomics/ 62666

17 (((system or systems) adj3 (science or research or factors or engineering or dynamic* or thinking or design or barrier* or facilitator*)) or "quality improvement" or "human factors" or ergonomics).ti,ab,kf. 160057

18 16 or 17 216740

19 6 and 15 and 18 742

EMBASE (1,509 results as of Nov 12, 2024)

Embase 1974 to present

1 exp antibiotic resistance/ 236633

2 ("antimicrobial resistance" or "antimicrobial stewardship").ti,ab,kf. 65199

3 amr.ti,ab,kf. 17578

4 exp antibiotic agent/ 2009800

5 antibiotic*.ti,ab,kf. 616160

6 1 or 2 or 3 or 4 or 5 2228775

7 exp United States/ 1435452

8 exp United Kingdom/ 488763

9 exp "Australia and New Zealand"/ 279502

10 exp Canada/ 229902

11 exp Europe/ 1888963

12 exp Norway/ 51393

13 ("united states" or "USA" or "United Kingdom" or "UK" or "Britain" or "England" or "Scotland" or "Wales" or "Northern Ireland" or "National Health Service" or "NHS" or "Australia*" or "Canada*" or "New Zealand" or "Europe" or "Norway").mp. 4414863

14 7 or 8 or 9 or 10 or 11 or 12 or 13 5651670

15 exp systems science/ 333

16 exp systems thinking/ 181

17 exp human factors research/ 1498

18 (((system or systems) adj3 (research or science or factors or engineering or dynamic* or thinking or design or barrier* or facilitator*)) or "quality improvement" or "human factors" or ergonomics).ti,ab,kf. 215022

19 15 or 16 or 17 or 18 215838

20 6 and 14 and 19 1565

**Grey literature**

Thesis/dissertation portals were searched on 15 February 2025 and included Open Access Theses and Dissertations, DART Europe, Open Grey, NDLTD.org, Trove, Theses Canada, Melinda Finland, Theseus Finland, Theses France, Deutsche National Bibliothek Germany, Google Scholar, Global ETD, Cybertesis, Bielefeld Academic Search Engine, Netherlands Research Portal, NZ Research, ProQuest, DiVA Scandinavia, Tesis Doctorales en Red Spain, Helveticat Switzerland, Tez Merkezi, and the UK’s ETHoS Metadata database.

**Appendix 3: Use of systems approaches and results**

| **Study** | **Date** | **How systems approaches were used** | **Results (as stated by the author)** |
| --- | --- | --- | --- |
| McNeil | 2017 | SEIPS was used to underpin the rationale of the study, inform the interview and focus group topic guides, and structure the data analysis. Authors clearly stated that system barriers interact and change over time and argued that this evolution is a key reason why understanding the context before intervention implementation is imperative for meaningful change. Focus groups, point prevalence surveys, semi-structured interviews, and an instant reporting system were used for data collection to understand system facilitators and barriers more robustly than with one approach alone. Specifically, authors used a focus group to identify barriers and facilitators from the perspectives of subject matter experts, point prevalence surveys to gather objective data about antimicrobial use, incident reporting system reports to understand real time barriers, and interviews with staff to understand their barriers and adaptations. Qualitative data were reviewed deductively using SEIPS then inductively to identify any codes not captured by the SEIPS framework. | The authors identified two key processes in antimicrobial management: collecting national indicator data and complying with the indicators. Barriers to data collection included low motivation, frequent staff turnover, poor app design, and lack of training, while facilitators included consistent nursing staff, organisational culture, and consultant involvement. Compliance with indicators was challenged by competing clinical priorities, documentation issues, workload, and variable knowledge, but was supported by feedback mechanisms, ward champions, and structured tools like antibiotic sections on kardexes. Compliance varied between wards, with indication more reliably documented than duration or review dates. Different system components were found across the medical and surgical wards, though all components of the SEIPS framework were identified to impact antimicrobial management. |
| Keller | 2018 | SEIPS was thoroughly explained in the Introduction and was used to inform the inclusion criteria, data collection, data extraction, and analysis. Findings from the articles reviewed were presented in SEIPS categories. SEIPS informed data collection in order to understand how the system factors impact processes, which impact outcomes. Data abstracted using components of the SEIPS 2.0 work system model, and its five components. | This review ultimately included 59 studies (42 quantitative (including randomised trials and quasi-experimental studies) and 17 qualitative) that evaluated ambulatory AMS interventions through the SEIPS 2.0 lens. Tools and technologies, especially EHR-based clinical decision support systems, showed promise but were often hindered by workflow and infrastructure challenges. Mixed effectiveness of educational and audit-feedback interventions, both of which were heavily influenced by context and implementation. Interventions were most effective when multifaceted and aligned with clinic workflows, patient expectations, clinician training, and broader organizational and societal structures. |
| Tarrant | 2021 | In the Introduction, authors described the importance of considering the social and structural context and its impact on individual prescribing behaviour. Authors also highlight examples of antibiotics being the 'quick fix' for larger structural challenges. Authors reported findings across three major themes: 1) Individual 2) Social and 3) Structural. Structural factors capture system factors. Authors explicitly stated in the Discussion section that there is more that needs to be done beyond behaviour change interventions to address the problem of AMR and state that "interventions targeting prescriber behaviour change risk 'individualising' the problem". Semi-structured interviews were conducted with the hospital prescribers across the seven hospital sites. The interviews sought to explore antibiotic prescribing decisions, the choice of broad-spectrum vs narrow-spectrum antibiotics including the benefits of this choice to various stakeholders, and perceptions of antimicrobial resistance. An inductive, constant comparative approach was used for the analysis. | Interviews were conducted with 46 prescribers (18 from Sri Lanka, 13 from South Africa, and 15 from the UK). Authors map out the extent to which different individual, social, and structural factors were major contributors to BSA overuse or were supporting factors by country and by hospital type (private or public). Authors found that the overuse of broad spectrum antibiotics is driven by a combination of individual-level factors (e.g., fear of diagnostic uncertainty and litigation), social dynamics (e.g., norms and hierarchies), and structural conditions such as limited diagnostic resources, patient poverty, and conflicting institutional incentives. |
| Safdar | 2021 | SEIPS will be used to 1) gain contextual site information, 2) understand implementation practices, and 3) assess the intervention. SEIPS will be used to link characteristics of each ICU to effectiveness outcomes. Authors opted to use SEIPS to develop a 'thick' description of the context associated with implementation at each ICU. Implementation process documentation, surveys, interviews, and focus groups were used for data collection. The extent to which SEIPS informed the development of these data collection tools is unclear. Authors emphasised that their analysis would focus on the "potential contributing factors that function as a system. Authors extensively described how SEIPS would be used to link context with outcomes in a visualisation of the SEIPS model. | No results-Protocol paper |
| Ramly | 2021 | The study applied a common model as a basis for comparison grounded in Rosen's modeling relation (1978). Rosen proposes that a model serves as a representation of the real world-system through mapping aspects of the system into key features. It has been applied in systems engineering to structure the description of clinician-patient interactions and constraints in the workflow. Semi-structured interviews with nurses and providers were used to understand normal work once a change in condition is noted (nurses) and factors that influence prescribing decisions (providers). Observations were used to inform process maps. Process maps and multi-site comparison matrices of workflow tasks were developed during analysis. Analysing and visualising data in this way enabled an understanding of variation in workflow processes within and between sites. | The authors propose and test a four step methodology for comparing clinical workflows across multiple sites and use antibiotic prescribing in 6 US nursing homes as a case study. They conducted one site visit and 44 individual or group interviews with 68 participants. They produced a generic workflow map and all 17 tasks from this workflow map were identified in at least half the sites with 10 present in all of the sites (staff workup tasks and nurse-provider communication tasks). Authors categorised each generic task as 'standard practice' (present in all sites in Generic form only), semi standard practice (present in all sites with majority Generic form only), and ubiquitous practice (present in all sites in Generic form and Variant form). Authors also categorised the percent of times each discipline (e.g., nurses, providers, etc) mentioned each task from the generic workflow during interviews as a proxy to understand the lowest mentions and therefore the areas where attention should be focused during improvements. |
| VanDort | 2024 | Authors stated that they used SEIPS given their aims of understanding the context of normal work by AMS teams. Authors aimed to enable a comprehensive approach to understanding the factors that impact AMS work. Observations and interviews were guided by the SEIPS framework. The interview topic guide was designed by the research team and was informed by SEIPS. The topic guide was piloted with doctors before use to ensure understanding. The data from the interviews and observations were coded inductively first, then the data were deductively mapped to the SEIPS framework. | This qualitative study compared antimicrobial stewardship (AMS) practices at two digital hospitals using the same electronic systems. Despite similar tools, Hospital A used the Live AMS dashboard more extensively, likely because of local involvement in its development and senior staff advocacy. Hospital B faced interoperability issues and lacked awareness or engagement with the tool. Organisational culture, executive support, and strong interdepartmental relationships were enablers of effective AMS. Hospital A showed more integrated, supported, and proactive stewardship activities and had more multidisciplinary meetings in which the tool was used. |
| Broom | 2021 | Authors extensively illustrate that existing forms of managerial accountability are often at odds with longer term health threats, such as AMR. Authors also describe that, while there are national guidelines to reduce AMR, it is unclear how these are infused within institutions. Authors reference several meso- and macro-organsational system factors that impact AMR. The interview topic guides included questions related to priority setting, normativity in what constitutes 'good', change management, accountability structures, and short- and long-term goals. The thematic analysis was guided by four questions from previous literature: 1) "What is the basis for a particular experience, action, belief, relationship, or structure?, 2) What do these assume implicitly or explicitly about particular subjects and relationships?, 3) Of what larger process is this action/belief and so forth a part?, and 4) What are the implications of such actions/beliefs for particular actors/institutional forms?" | The study found that hospital executives and managers in Australia often see antimicrobial resistance (AMR) as a low priority due to institutional accountability structures that favour short-term, measurable outcomes like budgets and KPIs. As a result, antimicrobial stewardship (AMS) is frequently treated as a “tick-box” requirement, leading to superficial or symbolic actions rather than meaningful change. This institutional environment fosters micro-improvements that satisfy managerial expectations but do little to address the systemic challenges driving AMR. |
| Leyenaar | 2019 | Systems theories did not emerge until during the data coding and analysis when authors identified similarities between their emerging findings and the Pettigrew Receptive Contexts to Organisational Change framework. This Pettigrew framework was then incorporated into data analysis and adapted based on the data collected. Hospitals in the collaborative were asked to implement a change package, which included clinical pathways and order sets and communication tools to promote use of narrow spectrum antibiotics. Data were collected from interviews with each hospital and a review of each hospital's project notes. A grounded theory approach was used "to identify emergent concepts". Grounded theory approach was initially used but the Pettigrew framework was identified part way through based on synergies in the resulting themes. | The mixed-methods study evaluated a QI collaborative aiming to increase narrow spectrum antibiotic use for CAP across 37 US hospitals. The researchers analysed 142 written reports and interviewed 30 site leaders. The collaborative achieved modest improvements, with prescribing rates rising to 43% in emergency departments and 59% on inpatient wards. Eight key, interrelated domains were identified as facilitators of successful interdepartmental QI, including leadership support, clinical champions, evidence alignment, national influences, collaborative culture, departmental structure, implementation strategies, and strong interdepartmental relationships. |
| Krukas | 2020 | Authors involved human factors experts in reviewing the reports. Authors represented their findings according to system-based themes. Authors identified vancomycin-related reports from a state-wide incident reporting system. Authors excluded safety reports in which system factors could not be identified so that their final sample included only reports with discernible system-based contributors. Descriptive analysis (including event type, harm score, patient demographics) was used. Qualitative analysis was performed via a grounded theory approach. | The study analysed 143 patient safety event reports involving IV vanc and found that most safety issues occurred during administration, monitoring, and ordering stages. The most frequent errors were dose omissions or delays, improper dosing, and monitoring failures (primarily due to therapy management, poor care coordination, and incomplete information flow). The persistence of these hazards highlights a significant gap between research and clinical practice, given that research on this topic already exists. |
| Gagnon | 2014 | Authors used a systems approach to assess effectiveness, efficiency, and financial feasibility of the program. Authors described a systems approach as the interaction between people, policies, and procedures. A systems approach was applied by identifying inputs, processes, and outputs and discussing how each interacted with the program as a whole. In the Discussion, the authors expand on their findings using a systems lens and argue that a systems approach encompasses both what goes well and what doesn't go well and contrasts this with other, commonly used methods such as Lean. Authors specifically highlight the need to move beyond "compartmentalized" approaches. Authors structured their data collection to compare findings from a multidisciplinary review of the program versus intradepartmental review of the program. Their rationale was that a systems approach beyond the department in isolation was likely to uncover more robust findings related to inputs, processes, and outcomes and ultimately help in the assessment of how the pharmacy program impacts, and is impacted by, other disciplines. | The use of a systems approach revealed 13 areas for improvement, rather than the five areas originally identified through internal pharmacy review alone. Key outcomes included granting pharmacists independent authority to order serum vancomycin levels and incorporating a dosing table into the institutional antibiotic guide. |
| Catho | 2020 | The Flottorp framework for determinants of clinical practice was applied to inform the interview guide. This includes seven areas determining practice, such as individual health professional factors, social, political, and legal factors, guideline factors, and potential for organisational change. Semi-structured interviews were conducted, with interview guide questions guided by a review of the literature and the Flottorp framework for determinants of clinical practice. Deductive (framework) analysis and an inductive approach. | This qualitative study identified key barriers to physicians' adherence to antimicrobial guidelines, including unclear or inaccessible guidelines, insufficient critical thinking skills, and hierarchical prescribing. It also found that adoption of computerised decision support systems (CDSSs) may be hindered by perceptions of increased workload, reduced autonomy, and medico-legal concerns (though physicians acknowledged the potential for CDSSs to improve guideline adherence and patient care if designed to be user-friendly, fast, and well-integrated into clinical workflows). Physicians also perceived that one size does not fit all and had difficulty accepting the rigidity of the guidelines for bespoke patient care needs. |
| Katz | 2017 | The Systems Engineering Initiative for Patient Safety (SEIPS) informed the study at every stage. The rationale for using SEIPS was provided in the introduction, SEIPS categories informed the data that was extracted from the articles review, and it served as a deductive framework for analysis and discussion. SEIPS was used to guide data extraction and analysis. SEIPS was selected because it can support the development and implementation of healthcare interventions by prompting the consideration of complex contextual factors related to long-term care facilities. | This integrative review analysed 20 studies (5 randomised controlled trials and 15 quasi-experimental studies) on antimicrobial stewardship in long-term care facilities (LTCFs) using a human factors framework. Effective interventions often combined structured, multidisciplinary education (included in 80% of studies), workflow-integrated tools, and infectious disease consultant involvement. Authors tracked the AMS interventions according to the following categories across each included paper: structured education, nurses included in the intervention, tools and technology, tasks, organisation, persons, environment, and measurable change. Several interventions led to sustained reductions in antibiotic use (e.g., one study reported a 30% reduction sustained over 30 months). |
| McLellan | 2016 | System factors were extensively discussed in the Discussion section, with substantial systems theory included to interpret and make meaning of the results. Authors extensively discussed that the system within which junior doctors operate is a complex, adaptive system, meaning that interventions cannot have "consistent and predictable effects". No explicit use of systems theory prior. Authors implemented a single-blinded, randomized controlled trial to understand the impact of a pharmacist feedback intervention on junior doctor prescribing. Qualitative and quantitative data were collected. Qualitative process evaluation data were collected via interviews. Whether any particular theory informed the interview topic guide is not explicitly stated but seems to be informed by behaviour change theory. Quantitative data included information about the prescription and prescription appropriateness. An inductive, constant comparative approach was used to analyse the qualitative data. | Pharmacist-led feedback workshops significantly improved antimicrobial prescribing among junior doctors. For the intervention group (n=14), there were 37 appropriate and 38 suboptimal prescriptions. For the control group (n=21), there were 51 appropriate and 78 suboptimal prescriptions. Improvements were largely due to better prescription writing, and qualitative data showed the intervention influenced behaviour by increasing awareness, reflection, and motivation within a complex social context. |
| Hernandez | 2024 | SEIPS was used to inform the interview topic guide. Authors stated that this was to understand the participant's role in the integration of the innovation into the complex work system. Less explicitly, authors alluded to complex sociotechnical systems in the introduction by stating that contextual factors, such as culture, must be considered for successful implementation. Authors also used the Urquhart framework, which aims to explore the role of middle managers in clinical implementation, to guide the study design. No further information about SEIPS (e.g., how it was used in analysis) is provided. Data were analysed inductively and deductively. The Urquhart framework was used to guide deductive analysis. | 4 Clinical Implementation Coordinators (CICs) across 4 sites were interviewed for the study.  CICs played a critical role in embedding a fluoroquinolone pre authorisation protocol in ICUs. CICs fulfilled roles as clinicians, planners, coordinators, facilitators, motivators, and evaluators. Key facilitators included antibiotic stewardship team empowerment and strong stakeholder relationships, while barriers stemmed mainly from staffing models and COVID-19-related disruptions. |
| Ramly | 2020 | While the study sought to not commit to a single theoretical view regarding antibiotic prescribing to support a naturalistic inquiry approach, the study design drew on the work systems model from human factors, considering the interdependency between health outcomes, care processes, individual healthcare providers, and the tools they apply within their organizational context. The Shannon-Weaver model of communication was also applied to represent the barriers related to aspects of information transmission. Observations and document reviews were used to inform the workflow analysis. Interviews were used to understand factors that influenced antibiotic use. Authors also collected documents and other written policies to inform the study ("artefact analysis"). There was no commitment to a priori theory to guide data collection. An inductive-deductive approach was used. The interactions between barriers identified were mapped using the Shannon-Weaver model of communication. This model was selected throughout the study based on emerging themes primarily related to challenges with communication of resident assessment across organisational boundaries. | This study identified key workflow barriers to appropriate antibiotic prescribing in nursing homes, including inconsistent nurse assessments, poor alignment of communication tools with clinical needs, difficulty reaching prescribers, and low mutual confidence between nurses and prescribers. These barriers were categorised into three themes: information (e.g., incomplete or inconsistent assessments), communication (e.g., mismatch with prescriber information needs), and professional challenges (e.g., variable prescriber confidence in nurse assessment report). The authors recommend integrated strategies (e.g., structured information tools, interprofessional education, and organisational support) to address these issues. Authors also mapped existing resources to help address the barriers in the three categories identified. |
| Aagaard | 2010 | In the Introduction, authors described the various system factors that influence physician prescribing patterns and described the importance of organisational context on implementation. Multidisciplinary focus groups and interviews were conducted. The rationale is not stated but it was likely used given that the authors had tried to understand the variability in uptake previously and needed to hear directly from those involved in doing the work. Inductive thematic analysis to ID OEMs. Once OEMs identified, specific hypotheses generated as to the impact of these on the effectiveness of the IMPAACT intervention.  This was developed into a qualitative rating scale, which was used to rate sites. | The IMPAACT study found that while a national educational intervention to reduce antibiotic prescribing for acute respiratory infections in EDs led to modest overall improvements, its effectiveness varied widely by site. The presence of a strong local physician champion emerged as the most critical factor influencing success but institutional emphasis on patient satisfaction and prior quality improvement history had less consistent impact. |
| Bugeja | 2020 | Authors extensively explained their rationale for using systems approaches (specifically SEIPS). Authors justified their approach by introducing principles such as emergence, human-environment interaction, and micro-, meso-, and macro- system interactions. Authors highlight that very few of their resulting articles made any explicit reference to human factors and, as a result, their extraction was largely subjective. Authors depicted a very high level, generic OPAT process map with six steps and used these six steps as rows of a matrix, with SEIPS factors serving as the columns in the matrix. Data extraction was informed by the SEIPS 2.0 categories, including the systems factors, interactions, and work processes. The data synthesis strategy consisted of three steps, all of which were informed by SEIPS 1) identification of work system factors, 2) identification of work processes and their category, and 3) identification of interactions between the work system factors. | This systematic review of 27 studies found that OPAT services achieve high treatment success rates and low readmission rates but improve satisfaction and reduce hospital associated infections (HAI). Key themes identified using the SEIPS 2.0 Human Factors model included the importance of standardised patient selection, staff and patient education, effective communication, and the usability of tools and technologies. |
| Valmadrid | 2021 | Authors cited systems theory in their rationale for the study and highlighted specific system barriers that may be amplified in populations with a number of care transitions. Minimal discussion of SEIPS otherwise. Authors specifically stated that SEIPS was selected to guide interview data collection because SEIPS can capture all elements of the work system and how they interact to influence outcomes. Authors overtly stated that SEIPS did not guide the analysis. Authors used an inductive and deductive approach iteratively throughout coding. | 32 staff members of long-term care facilities (LTCFs) and EDs were interviewed. The study found that fragmented communication and power dynamics between emergency department (ED) and LTCF providers contribute to inappropriate antibiotic use for suspected urinary tract infections (UTIs) in older adults. Nurses play a role in both assessing patients and relaying information that influences prescribing decisions, yet communication challenges (especially across care settings) can lead to overtreatment. To address this, strategies such as communication scripts, nurse education, improving longitudinal relationships, and standardised diagnostic tools were identified to support more effective antibiotic stewardship. |
| Knobloch | 2021 | SEIPS was used to inform the study design, specifically to inform data collection related to workflow and organisational factors that influence nurse practitioner decision making. Inductive and deductive approaches were used to analyse nurse practitioner interview findings. The patient focus groups were also informed by SEIPS domains. Findings from both the nurse practitioner interviews and patient focus groups were integrated and mapped onto the SEIPS categories. | 14 nurse practitioners (NP) were interviewed and focus groups conducted with 15 veterans. NP prescribing data was reviewed for 2017-2019. Over 3 years, there was a downward trend in the proportion of antibiotics prescribed by VA outpatient NPs, which was aligned with when NPs were given increased prescribing autonomy. Qualitative findings revealed that NPs valued access to pharmacists and decision support tools, emphasised patient education, and cited patient-provider trust as key to guideline-concordant prescribing. |
| Kianmehr | 2020 | Authors justified the need for their study in the Introduction by stating that interactions between social factors can contribute to overprescribing. The system dynamics simulation model results were displayed to illustrate the relationship between the feedback loops in the system (e.g., the influence of provider's perception of patient expectation on the provider's decision effectiveness in identifying the patient's actual expectation). A system dynamics simulation modelling methodology was used due to its appropriateness in studying dynamic behaviours in complex systems and interrelated causal loops between variables in a system. | The study developed a system dynamics model to simulate antibiotic prescribing behaviours for acute respiratory tract infections (ARTIs) in the US, and validated the model for different age groups and regions via the National ambulatory Medical Care Survey database.  Results indicated that t prescribing decisions are influenced by physician’s experience and prior prescribing behaviour, patient expectations, providers’ perceptions of those expectations, and perceived infection risk. Simulation results indicate that patient expectations have declined over time and that regional and age-related variations exist in prescribing trends. |
| Carayon | 2021 | The data collection and analysis were guided by the Systems Engineering Initiative for Patient Safety (SEIPS) and evidence-based implementation principles. These frameworks were used to describe the different aspects of implementation of the FQ PPA intervention.  1. Semi-structured interviews were conducted with two key informants at the two hospitals who were active leaders for the design and implementation of the intervention.  2. Implementation diaries following a structured template were completed by the key informants before, during, and after the intervention went live.  3. Meeting notes were collated from regular meetings with the key informants before and after the intervention implementation.  4. Document analysis of key documents was undertaken to review contextual data and information on the design and implementation of the intervention at each site.  5. Screenshots of 'Best Practice Alerts' (BPA) in the Electronic Health Record and discussions with key informants were used to document clinician workflow when encountering the BPA. *Analysis:* SEIPS model, which focussed on five work system elements and to describe the FQ PPA workflow. Systematic qualitative data analysis of interview transcripts was performed. Excerpts were coded based on the following dimensions  1) specific implementation principles  2) whether excerpt referred to before or after the project went live  3) whether the excerpt was a barrier or facilitator of implementation. | The fluoroquinolone pre-prescription authorisation (FQ PPA) intervention led to reduced FQ use in both ICUs, measured in days of therapy per 1,000 patient-days. At Site A, FQ use declined consistently post-implementation, while Site B showed a downward trend even before the intervention due to proactive education and engagement. Site A permitted only 3 approved indications for FQ use while site B included 10 approved indications. Leadership commitment, user participation, communication, training, organisational learning, and project management were key for implementation. |
| Pulia | 2022 | The study applied the SEIPS framework to guide their data collection and analysis approach. Probing follow-up questions following the elements of the framework were included in the interview guide, and barriers and facilitators to optimal prescribing were identified based on the elements of the framework. SEIPS was used as it has been previously applied to characterize quality and patient safety challenges and includes a person-centred perspective with surrounding interacting elements. Semi structured interviews were used to explore themes around the diagnostic and antibiotic decision-making process for skin and soft-tissue infections. The initial questions were open-ended to enable participants to share their initial reactions, followed by probing questions aligned to the SEIPS framework. The data were analysed using deductive directed content analysis guided by the SEIPS framework to identify the barriers and facilitators to optimal antibiotic prescribing. A list of proposed interventions was matched to the identified barriers and enablers. | 20 interviews were conducted with physicians in EDs. Poor access to follow-up care, diagnostic uncertainty (especially for cellulitis and MRSA), patient expectations, high risk patient profile, prioritisation of proximal complaint over potential consequences of antibiotics, lack of diagnostic tests, emphasis on patient satisfaction scores, equipoise in the literature, and provider fear of adverse outcomes were identified as barriers to optimal prescribing. Facilitators included shared decision making conversations, identifying as an antibiotic steward, considering cellulitis mimics, watchful waiting, and wait and see prescribing. |
| Legenza | 2023 | In the Introduction, authors described the usefulness of Cognitive Task Analysis (CTA) as a method to understand decision making, particularly in high pressure situations. Authors included an extensive appendix outlining their rationale for a hierarchical task analysis (HTA) and operations sequencing diagramming (OSD) in their methodology. Per the authors, each method offered different insights and complemented shortcomings in the others (e.g., HTA focuses on the goals of each step while OSD focuses on the sequence of the steps and the interactions between tasks that are not immediately visible). Data collection focused on provider workflows, verbalised decision making, availability of resources, and interactions with other patients/providers. Brief interviews were conducted during the observations (e.g., between patients). Inductive content analysis was used and themes were arranged to develop the HTA and OSD. The drafts of both models were shared back with the participants for member checking. | The study observed 78 patient-provider encounters across 39 hours in rural family medicine, urgent care, and emergency settings. Key themes included the cognitive complexity of clinic visits, the variability introduced by time pressures, and the importance of shared decision-making (especially when treatment options are ambiguous). Antibiotic prescribing was relatively infrequent, and providers rarely used local antimicrobial resistance data, instead relying on general resources like UpToDate. The findings stress the need for clinical decision support tools that are efficient, context-aware, and well-integrated into the provider’s workflow. |
| Hughes | 2024 | Authors described the limitations of studying healthcare systems through a linear lens and introduced the importance of complexity theory based on this rationale. A definition was provided for complexity theory and this definition, as well as characteristics of complexity theory, explicitly guided their data collection. Authors included only articles that explicitly used complexity theory to understand AMS in healthcare. Authors included an extensive table of characteristics of what was meant by 'complexity theory' to inform inclusion/exclusion. Authors extracted data including the setting, aims, study population, methods, intervention, outcomes/key findings, and how complexity theory was discussed in the article. Only 8 total articles were included so analysis between articles was minimal. Additionally, many articles included in this review were from countries around the world, rather than high-income countries specifically. | 8 articles were ultimately included (i.e., only eight studies explicitly used complexity theory to inform antimicrobial stewardship (AMS)) with considerable variation in how the theory was applied and operationalised. Authors highlighted only two major themes from the 8 articles reviewed: 1) importance of tailored communication strategies and 2) use of mixed methods. |
| Van Dort |  | SEIPS was used given the study's aims of understanding how diverse clinical environments impacted AMS. The study took a contextual inquiry approach through a mix of observations and semi-structured interviews guided by SEIPS with AMS team members. Data were initially analysed using an inductive content analysis approach, after which the codes were deductively mapped based on the SEIPS 2.0 model. Authors triangulated their observational data ('what they were seeing') with their interview data ('why these practices happened according to the participants') | Authors conducted 11.5 hours of observations in Hospital A and 16 hours of observations in Hospital B with 4 total interviews. Authors mapped the barriers and facilitators within the interactions between each SEIPS domain (e.g., barriers were reported within the technology-task interaction). Technology was identified to be a 'double edged sword', in the sense that it enabled some improved communication and efficiency, but was hindered by interoperability challenges. Organisational factors, physical presence and face-to-face interactions were also identified as critical factors for the execution of AMS strategies. Finally, individual staff members, particularly the role of ID consultants, and their relationships played a pivotal role. |

**Appendix 4: Articles excluded at the full text screening stage**

Seventy six of the 92 articles excluded at the full text screening stage were articles in which system factors were opportunistically identified but a systems approach was not used, and were therefore excluded from this review. There were three primary reasons these articles were not included (Examples are included in the referenced citation):

1. Articles identified system factors but the use of systems approaches was never explicitly stated[^1,2^](https://www.zotero.org/google-docs/?921iDx)
2. Articles identified system factors that influenced antimicrobial use but the approach used was based in another, similar discipline, such as behaviour change[^3^](https://www.zotero.org/google-docs/?WeAMKy)
3. Articles focused on one system factor in isolation (e.g., organisational factors) rather than the interactions between these factors.[^4^](https://www.zotero.org/google-docs/?k2P5dI)

Many of these articles should have been excluded for more than one reason but only one reason could be selected. Other articles made it to the full text screening but were excluded for other reasons. See the attached Excel sheet for a full list of articles excluded at the full text screening stage and the reasons for their exclusion.

Reference List

[1. Harbin NJ, Lindbæk M, Romøren M. Barriers and facilitators of appropriate antibiotic use in primary care institutions after an antibiotic quality improvement program – a nested qualitative study. *BMC Geriatr*. 2022;22(1):458. doi:10.1186/s12877-022-03161-w](https://www.zotero.org/google-docs/?vM2vn9)

[2. Dallas A, Driel M van, Mortel T van de, Magin P. Antibiotic prescribing for the future: exploring the attitudes of trainees in general practice. *Br J Gen Pract*. 2014;64(626):e561-e567. doi:10.3399/bjgp14X681373](https://www.zotero.org/google-docs/?vM2vn9)

[3. May L, Gudger G, Armstrong P, et al. Multisite Exploration of Clinical Decision Making for Antibiotic Use by Emergency Medicine Providers Using Quantitative and Qualitative Methods. *Infect Control Hosp Epidemiol*. 2014;35(9):1114-1125. doi:10.1086/677637](https://www.zotero.org/google-docs/?vM2vn9)

[4. Aspinall SL, Berlin JA, Zhang Y, Metlay JP. Facility-level variation in antibiotic prescriptions for veterans with upper respiratory infections. *Clin Ther*. 2005;27(2):258-262. doi:10.1016/j.clinthera.2005.02.002](https://www.zotero.org/google-docs/?vM2vn9)
