# Supplementary material for: The latent system factors that influence antimicrobial use and governance in healthcare: a scoping review of high-income health systems
Source: eClinicalMedicine. 2025 Sep 19;89:103520. doi: 10.1016/j.eclinm.2025.103520 (PMC12495420; doi:10.1016/j.eclinm.2025.103520)
Supplement: Multimedia component 2 [file mmc2.docx]

Dear Editors of *eClinical Medicine*,

We are pleased to submit our manuscript, titled "*The latent system factors that influence antimicrobial use and governance in healthcare: A scoping review”* for consideration*.* This review builds directly on the personal view article we recently published in your journal titled “*Designing better systems to navigate the sepsis-antimicrobial stewardship tension*”. In that personal view article, we outlined the urgent need to understand the system factors not captured in policies and procedures that shape antimicrobial use in real-world healthcare settings. The present scoping review is a direct follow-up to that call to action and offers a comprehensive synthesis of how systems approaches have been used in existing antimicrobial stewardship (AMS) research.

This scoping review addresses a critical gap in our current understanding of a major global priority: while judicious and timely use of antimicrobials can minimise sepsis-related harm, the systems in which they are prescribed often undermine stewardship efforts. Clearly, there is a gap between the best practice evidence and what happens in reality. System factors, such as competing pressures, process ambiguities, incompatible clinical tensions, and demand-capacity misalignments, continue to drive this gap.

In this scoping review, we map how systems approaches have been applied to understand antimicrobial use and governance in healthcare settings. The relatively small number of studies included in this review suggests that there has been little application of systems approaches, even in countries with well-established healthcare systems. Additionally, we found that, of the studies that have applied systems approaches, most are limited to the micro level (i.e., direct patient care), with far fewer investigating the meso (management) or macro (leadership) levels, despite their critical role in coordination, implementation, and strategic decision making. **This suggests a persistent blind spot for AMS improvement.** Specifically, the downstream challenges clinicians face are often rooted in upstream system factors that remain poorly understood and understudied.

Analysis of system factors that influence use of antimicrobials within direct patient care is imperative. However, frontline challenges are significantly influenced by the institutionally-sanctioned context within which they occur and are often downstream consequences of system factors at higher organisational levels. The limited application of systems approaches beyond the frontline level suggests a gap in understanding how organisational alignment toward the goal of AMS-or lack thereof- emerges across levels due to system factors within and between levels.

**We believe that this scoping review will be of significant interest to your audience specifically because**

- To our knowledge, this is the first review to construct a new understanding of the implications of systems approaches for AMS at micro, meso, and macro organisational levels
- It builds on the previously published personal view by highlighting what is actually known and unknown in the literature, which provides a shared starting point for future work and addresses an urgent implementation gap
- By highlighting how systems approaches can uncover hidden facilitators and barriers to antimicrobial use, our review offers practical insights to increase the impact of AMS interventions in complex, dynamic healthcare settings.

We are excited to submit this manuscript for your consideration and believe it will make a timely and significant contribution to the literature regarding antimicrobial resistance, sepsis, and infectious diseases more broadly. Thank you for considering our submission.

Thank you

Olivia Lounsbury, MS*

Nuffield Department of Clinical Neurosciences, University of Oxford

Headley Way, Headington, Oxford OX3 9DU

*Corresponding author [olivia.lounsbury@ndcn.ox.ac.uk](mailto:olivia.lounsbury@nds.ox.ac.uk)

(01865) 231510

Marta Ortega Vega, BSc

Florence Nightingale Faculty of Nursing, Midwifery & Palliative Care, Methodologies Division, King’s College London, UK

Lauren Hookham, MBBS

Department of Global Health and Infection, Brighton and Sussex Medical School, UK

Professor Jane O’Hara, PhD

The Healthcare Improvement Studies (THIS) Institute, University of Cambridge, UK; National Institute for Health and Care Research (NIHR) Yorkshire & Humber Patient Safety Research Collaboration (PSRC), Bradford, UK

Natalie Sanford, PhD

Florence Nightingale Faculty of Nursing, Midwifery & Palliative Care, Methodologies Division, King’s College London, UK

Kelly Williams, MSN

Patient Safety and Quality Department, The Johns Hopkins Hospital, USA

Professor Andrew J. Brent, FRCP

Chief Medical Officer & Professor of Infectious Diseases & General Medicine, Oxford University Hospitals NHS Foundation Trust, UK; Visiting Professor of Infectious Diseases & General Medicine, Nuffield Department of Medicine, University of Oxford, UK

Helen Higham, DPhil

Anaesthetics, Oxford University Hospitals NHS Foundation Trust; Oxford Simulation, Teaching, and Research Centre (OxSTaR), University of Oxford, UK
